# Evaluation of Mitochondrial Complex 1 Density with [^18^F]BCPP-EF in a Murine Model and Individuals with Friedreich Ataxia

**DOI:** 10.2967/jnumed.124.268698

**Published:** 2025-09

**Authors:** Laigao Chen, Gaia Rizzo, Christine Bulawa, Koene R.A. Van Dijk, Erica C. Henning, Alain Martelli, Jeffrey Palmer, Avery McIntosh, Marko Pregel, Pengling Sun, Emmanuel Adewunmi, Mark Aldridge, Jackson Chan, Roger N. Gunn, Mickael Huiban, Allan Listanco, Peter T. Loudon, Sara Moz, Jan Passchier, Lauren Sauvage, Rachel Stewart, Lisa Wells, Eugenii A. Rabiner, Lawrence R. Charnas, Richard J. Festenstein

**Affiliations:** 1 Pfizer, Cambridge, Massachusetts;; 2 Invicro, London, United Kingdom;; 3 Imperial College London, Hammersmith Hospital Campus, London, United Kingdom;; 4 Imperial College Healthcare Trust, Hammersmith Hospital, London, United Kingdom;; 5 Invicro, Needham, Massachusetts;; 6 Department of Brain Sciences, Faculty of Medicine, Imperial College London, Hammersmith Hospital Campus, London, United Kingdom; and; 7 Peter Loudon Consulting Ltd., Cambridge, United Kingdom

**Keywords:** [^18^F]BCPP-EF, frataxin, Friedreich ataxia, mitochondrial complex 1, PET

## Abstract

Friedreich ataxia is caused by mutations in the frataxin gene, leading to neurodegeneration and premature death from cardiac dysfunction. Loss of frataxin impacts mitochondrial complex 1 (MC1) activity, suggesting MC1 may be a potential biomarker of frataxin levels and function. Biomarkers evaluated by noninvasive techniques are needed to monitor disease progression and treatment effects in people with Friedreich ataxia. **Methods:** PET with [^18^F]BCPP-EF, a ligand with high binding specificity for MC1, was used to measure cardiac and brain MC1 density in a mouse model of Friedreich ataxia and in healthy volunteers and participants with Friedreich ataxia. **Results:** An imaging protocol was developed in humans that included a 70-min brain scan immediately after administration of [^18^F]BCPP-EF followed by a 60-min cardiac scan 255 min after [^18^F]BCPP-EF administration. Cardiac [^18^F]BCPP-EF binding in participants with Friedreich ataxia was lower than that in healthy volunteers and in a mouse model of Friedreich ataxia versus wild-type mice (∼50% reduction in both). In the brain, no statistically significant difference in the [^18^F]BCPP-EF binding was detected between participants with Friedreich ataxia and healthy volunteers. Correlation analyses showed that blood frataxin and cardiac [^18^F]BCPP-EF levels decreased with increasing guanine–adenine–adenine expansion size (*R* = −0.82 and −0.78, respectively; both *P* < 0.05) but not in the precentral gyrus (*R* = 0.63; *P* < 0.05). **Conclusion:** MC1 density as measured using [^18^F]BCPP-EF–based PET may be a viable biomarker of mitochondrial deficit and frataxin levels in people with Friedreich ataxia.

## Visual Abstract

**Figure:**
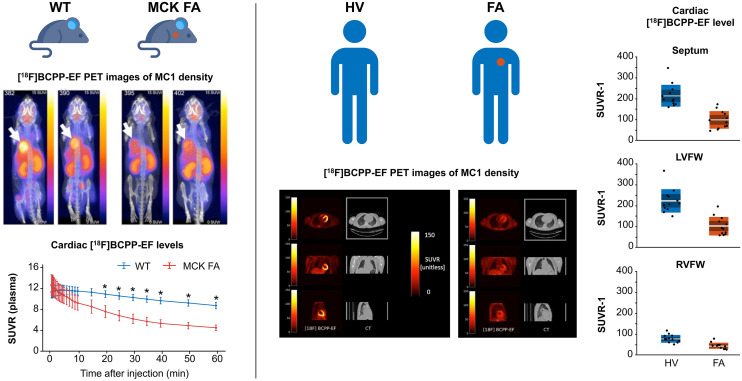

Friedreich ataxia (FA) is an autosomal recessive disease caused by partial deficiency of the mitochondrial protein frataxin, with most neuropathologic changes causing ataxia limited to the infratentorial sensory system and cerebellar dentate nuclei (1). Loss of Betz cells in the primary motor cortex and corticospinal tract degeneration may explain the clinical upper motor neuron findings (1). FA affects approximately 1 in 50,000 people in the United States and Europe (1–3). No noninvasive in vivo measurements of frataxin levels in tissues of interest are currently available. This would be a desirable readout for therapies designed to upregulate or replace frataxin or for use as a disease progression biomarker in individuals with FA. Frataxin deficiency leads to disrupted iron–sulfur cluster synthesis, resulting in downstream effects on mitochondrial complex 1 (MC1) activity and multiple other mitochondrial functions (4–9), suggesting MC1 activity could be a robust biomarker of frataxin levels and function.

Preliminary investigations identified several radiotracers that bind to MC1 and can be detected using PET (10,11). Further refinements led to the development of [^18^F]BCPP-EF, which demonstrated high binding specificity for MC1 in rodents (12). Subsequently, [^18^F]BCPP-EF PET was used to image the brains of rodents, nonhuman primates (12–14), healthy volunteers (HVs) (15,16), and clinical populations (17,18). To the best of our knowledge, [^18^F]BCPP-EF has not previously been applied to rodent models of FA and individuals with FA, and its utility for the assessment of MC1 in the human heart has not previously been evaluated.

The current research project had 4 aims: (i) to test whether [^18^F]BCPP-EF PET imaging can assess differences in MC1 density in cardiac and skeletal muscle from a conditional knockout mouse model of FA, (ii) to develop a protocol for the quantification of MC1 density in the human heart using [^18^F]BCPP-EF PET imaging, (iii) to compare the density of MC1 in the heart and specific brain regions between HVs and participants with FA, and (iv) to explore the relationship between MC1 density in the heart and brain as measured by [^18^F]BCPP-EF PET and the clinical characteristics of participants with FA.

## MATERIALS AND METHODS

### Ethics and Approvals

Procedures involving mice were conducted following Invicro’s standard operating procedures and Massachusetts Department of Public Health regulations. The protocol was reviewed and approved by Invicro’s Institutional Animal Care and Use Committee according to the Guide for the Care and Use of Laboratory Animals (19). Procedures in HVs and participants with FA were conducted in accordance with International Council for Harmonisation Good Clinical Practice and the Declaration of Helsinki. The study was approved by the London–Central Research Ethics Committee (reference: 19/WA/0070; IRAS: 257578). Permission to administer radioisotopes to humans was obtained from the Administration of Radioactive Substances Advisory Committee of the U.K. (reference: 538). All participants provided written informed consent before participation in the study.

### Muscle Creatine Kinase (MCK) FA Mouse Model

Generation of the MCK conditional *Fxn* knockout (MCK FA) mouse model has been described previously (20). Twelve mixed (1:1 male/female) MCK FA mice and 12 mixed wild-type (WT) mice of approximately 4 wk of age were obtained from The Jackson Laboratory (order number 029720). Animals were acclimated for 48 h and given food and water ad libitum for the duration of the study. Six WT and 6 MCK FA mice were imaged with [^18^F]BCPP-EF PET dynamically for 60 min, followed by γ-counting of various tissues. Details of experimental procedures and statistical analyses are in the supplemental materials (available at http://jnm.snmjournals.org).

### Procedures in HVs and Participants with FA

Individuals were recruited and screened at either the Clinical Research Facility at Imperial College (London, U.K.) or at Invicro (London, U.K.). All imaging procedures were conducted at the Invicro imaging center in London. A total of 18 HVs and 12 participants with FA were enrolled. Requirements for inclusion in the study are described in the supplemental materials.

Scanning protocols, including initial acquisition, protocol optimization, and final cardiac and brain protocols, are described in detail in the supplemental materials. Initial studies in 6 HVs evaluated the quantification of [^18^F]BCPP-EF in the human heart using a 120-min acquisition protocol (Supplemental Fig. 1A). To evaluate the test–retest variability of the method, all 6 participants were scanned twice with [^18^F]BCPP-EF PET, separated by approximately 2 wk. To optimize the protocol for estimating MC1 cardiac density, a variety of different procedures for scan times of up to 7 h were tested in 6 HVs (Supplemental Fig. 1B) before the optimal protocol was established as a one-time 60-min static cardiac scan starting 255 min after tracer injection (Supplemental Fig. 1C). Cardiac MC1 density in 12 HVs and 12 participants with FA was evaluated using this protocol. For the evaluation of brain MC1 density, the same 12 participants with FA underwent a one-time 70-min dynamic brain scan starting immediately after radiotracer injection (Supplemental Fig. 1D). Participants had a break before the cardiac scan was started at 255 min after injection. Data from 12 nonconcurrent age- and sex-matched HVs from the MIND MAPS database (15) were used for comparison of brain MC1 density.

### Analysis Methods and Kinetic Modeling in HVs and Participants with FA

Details of the analysis methods and kinetic modeling are given in the supplemental materials. Kinetic models were based on those previously described (21). Regions of interest are shown in Supplemental Figure 2.

### Frataxin Protein Measurement

Blood frataxin protein levels were quantified using the Frataxin Protein Quantity Dipstick Assay Kit (Abcam ab109881; Abcam Inc.). Blood samples were processed according to the manufacturer’s guidelines. Further details are available in the supplemental materials. This assay detects all frataxin, including in erythrocytes, which has been shown to be downregulated in people with FA to a similar extent as frataxin found in buccal cells and peripheral blood mononuclear cells (22,23).

### Statistical Analysis in HVs and Participants with FA

See supplemental materials for details.

## RESULTS

### Cardiac MC1 Density Is Lower in MCK FA Mice Than in WT Mice

**FIGURE 1.**
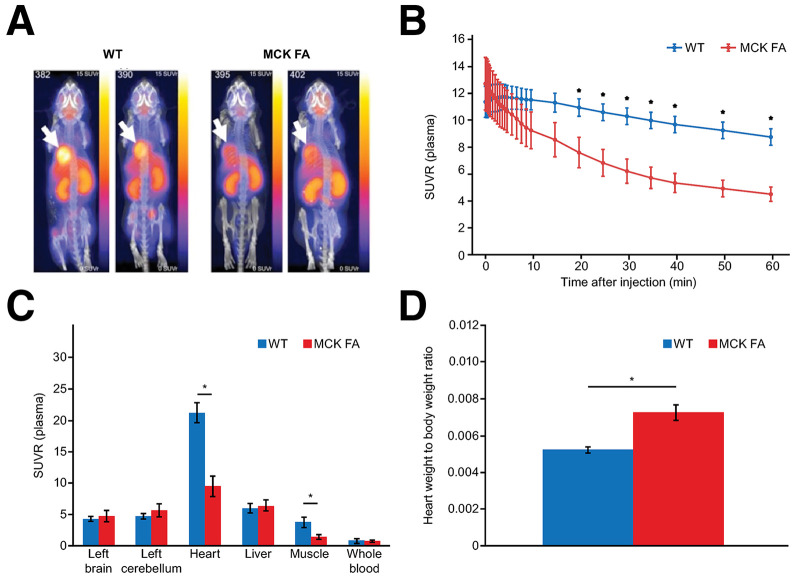
[^18^F]BCPP-EF PET imaging in WT and MCK FA mice. (A) Whole-body PET images of WT (left) and MCK FA (right) mice at 40–60 min after tracer injection; scaled 0 to 15 SUVR (relative to terminal plasma). Arrows indicate heart in each case. (B) Mean ± SEM cardiac [^18^F]BCPP-EF SUVR (relative to terminal plasma) in WT (*n* = 6) vs. MCK FA (*n* = 5) mice. (C) Ex vivo [^18^F]BCPP-EF distribution (SUVR relative to terminal plasma) in tissues for WT (*n* = 6) vs. MCK FA (*n* = 5) mice. (D) Mean ± SEM heart weight normalized to body weight for WT (*n* = 6) vs. MCK FA (*n* = 5) mice at ∼7 wk of age. *P < 0.05.

Representative images from WT and MCK FA mice (20) depicted reduced cardiac uptake of [^18^F]BCPP-EF in the MCK FA mice (Fig. 1A). SUV ratio (SUVR) time–activity curves revealed significantly lower [^18^F]BCPP-EF signal in the heart of MCK FA mice beginning at 20 min after tracer injection (*P* < 0.05; Fig. 1B). Ex vivo assessments showed significantly lower [^18^F]BCPP-EF SUVR in the heart and skeletal muscle of MCK FA mice compared with WT mice (∼50% reduction; *P* < 0.05; Fig. 1C), with no significant differences in the brain, cerebellum, liver, and whole blood, consistent with the knockout of frataxin only in heart and skeletal muscle. MCK FA mice had a significantly higher heart weight–to–body weight ratio compared with WT mice (mean ± SEM, 0.0072 ± 0.0004 vs. 0.0052 ± 0.0001, respectively; *P* < 0.05; Fig. 1D), consistent with cardiac hypertrophy.

### Protocol Development of MC1 Cardiac Density by [^18^F]BCPP-EF PET in HVs

**FIGURE 2.**
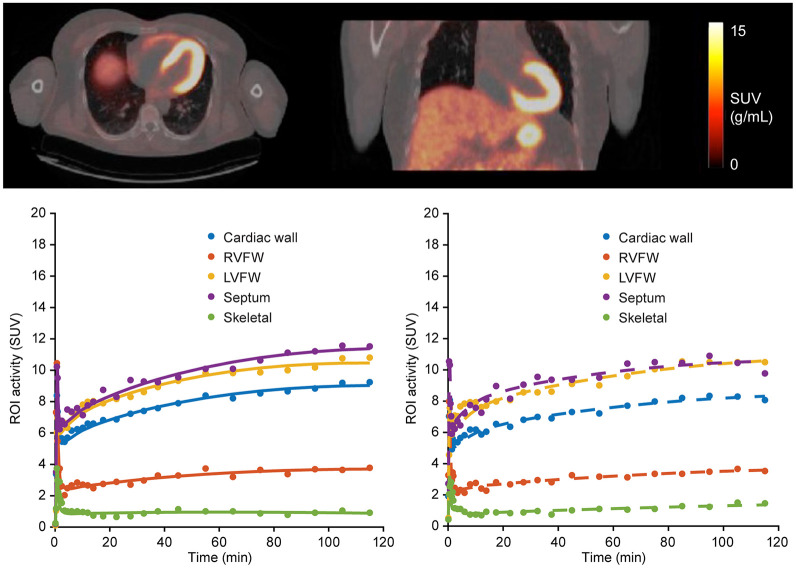
Feasibility of cardiac [^18^F]BCPP-EF PET imaging using 120-min acquisition. Top: orthogonal cross-section images of coregistered PET and CT images from HV. PET images are shown as SUV summed from 80 to 120 min after tracer injection. Bottom: SUV time–activity curves (circles) and one compartmental model fits (solid and dashed lines for test [left] and retest [right] scans, respectively) for different cardiac regions from HV. ROI = region of interest.

Cardiac [^18^F]BCPP-EF PET images from a representative participant are shown in Figure 2A. High PET signal was seen in the septum and left ventricle free wall (LVFW), followed by right ventricle free wall (RVFW). A one-tissue reversible compartment model with a fitted blood-volume distribution optimally described the kinetics in the heart. Time–activity curves and model fits from a representative participant are shown in Figure 2B. On assessment of outcome parameters, the influx rate was found to be approximately 100-fold higher than the efflux rate, indicating that the tracer was close to being flow-limited in the heart with the 120-min acquisition protocol. Volume of distribution (*V*_T_) test–retest data showed moderate to high test–retest variability (septum: 39% ± 37%; LVFW: 45% ± 15%; RVFW: 67% ± 36%). The 120-min acquisition protocol was therefore not considered suitable to evaluate cardiac MC1 density.

### Quantification of MC1 Cardiac Density Using [^18^F]BCPP-EF in HVs

**FIGURE 3.**
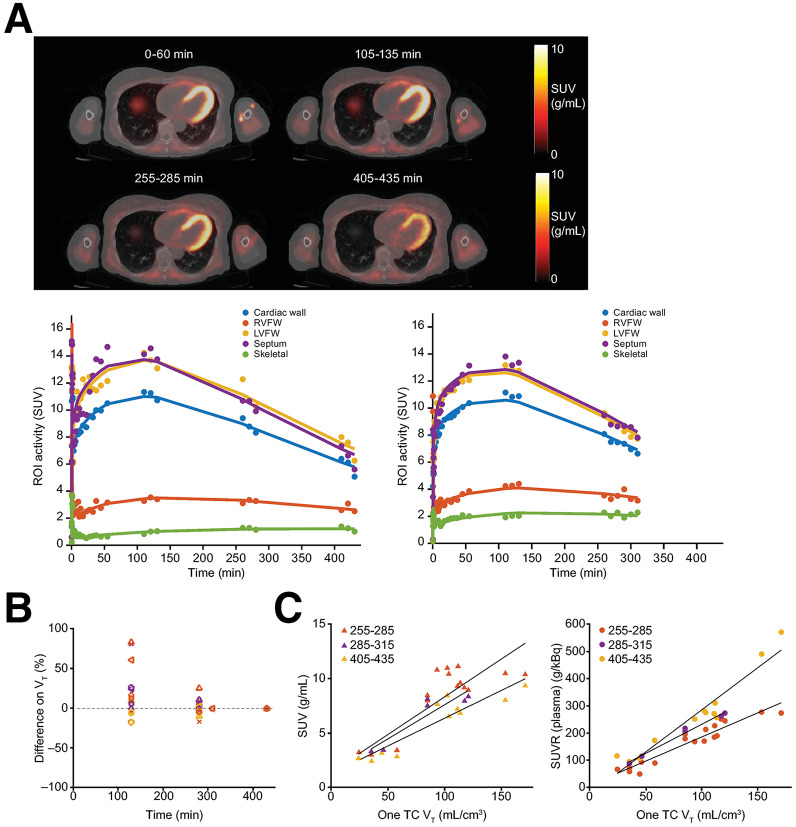
Estimation of cardiac MC1 expression using [^18^F]BCPP-EF PET. (A) Top: orthogonal cross-section images of coregistered PET and CT images from HV. PET images are shown as SUV summed from 0 to 60, 105 to 135, 255 to 285, and 405 to 435 min. Bottom: SUV time–activity curves (circles) and 1 compartmental model fit (solid lines) for different cardiac regions from 2 HVs. Individual in left panel underwent 4 scans up to ∼7 h after tracer injection. Individual in right panel underwent 3 scans up to ∼4 h after tracer injection. (B) Differences in cardiac *V*_T_ between data derived from full time course (435 or 315 min after tracer injection) and *V*_T_ derived from shorter scan intervals. Data for individual participants are represented by different shapes. Purple, septum; yellow, LVFW; red, RVFW. (C) Correlation of SUV and SUVR (plasma) vs. *V*_T_ from 1 compartmental model for various time intervals after tracer injection (red, 255–285 min; purple, 285–315 min; yellow, 405–435 min). Septum, LVFW, and RVFW are included in plots. ROI = region of interest; TC = tissue compartment.

To address the limited kinetic information and absence of washout data provided using the 120-min acquisition protocol, 6 HVs underwent up to 4 PET data acquisitions spaced up to 7 h after tracer injection to quantify cardiac MC1 density. Images from a representative participant are shown in Figure 3A. Time–activity curves were generated successfully, and reversible tissue kinetics were identified for all scans. A one-tissue reversible compartment model with a fitted blood-volume contribution was the optimal model to describe [^18^F]BCPP-EF kinetics. The model produced acceptable fits to tissue SUV time–activity curves in all cases (Fig. 3B). The *V*_T_ parameters derived from the model displayed high precision in all cardiac regions (coefficient of variation ranged from 1% to 5% for septum and LVFW and up to 20% for RVFW). Using time stability analysis, a minimum of 285 min of dynamic data were necessary to determine *V*_T_ accurately (Fig. 3C). At 4 h after tracer injection, the mean ± SD percentage differences of *V*_T_ in relation to those estimated from the full dataset were 3.7% ± 5.2% in the septum, −2.0% ± 5.2% in the LVFW, and 0.6% ± 14.2% in the RVFW.

To assess the feasibility of using static PET imaging to measure cardiac MC1 density, SUVR normalized against radioactivity in plasma attributed to the parent compound of the radiotracer was calculated at various intervals after injection. These values correlated well with *V*_T_ from the model in the interval of 255–285 min (*R* ∼ 0.87) and 405 to 435 min (*R* ∼ 0.96) (Fig. 3D).

### Comparison of MC1 Density in the Heart and Brain Between HVs and Participants with FA

**FIGURE 4.**
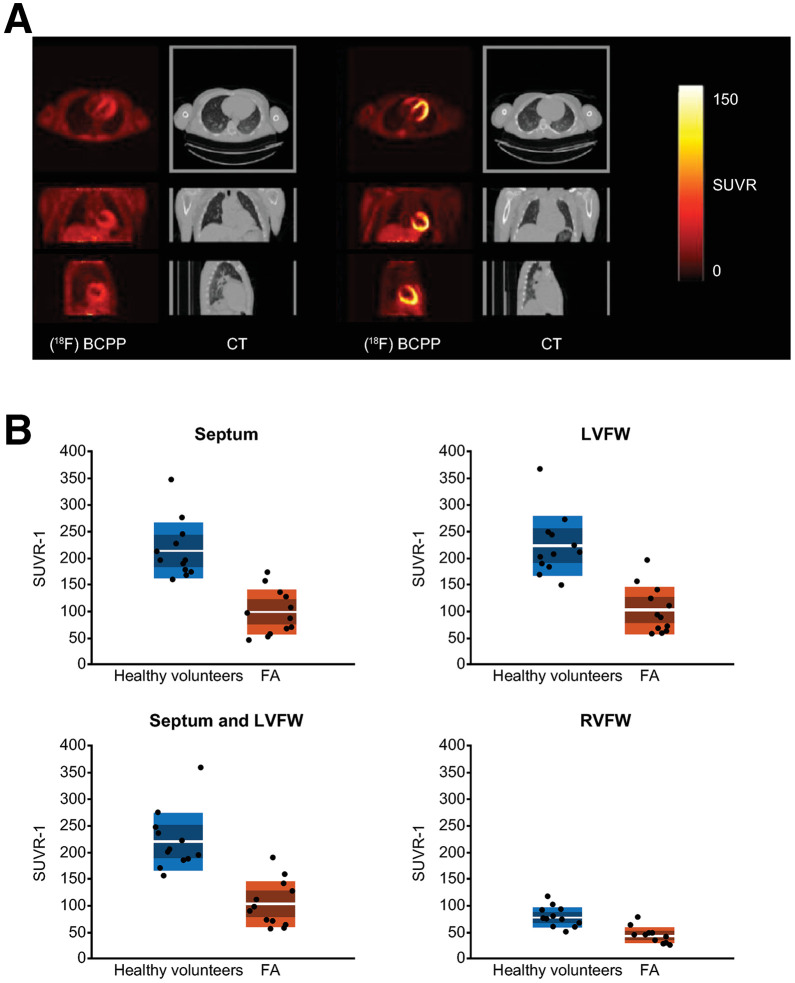
Comparison of cardiac MC1 density in HVs and participants with FA. (A) Orthogonal cross-section images of coregistered PET and CT images from individual with FA (left) and HV (right). Images are shown as SUVR (plasma) summed from 255 to 285 min after tracer injection. (B) Boxplots of SUVR−1 values in HVs (blue) and participants with FA (red).

The SUVR−1 (using parent plasma concentrations as a reference) over the period of 255–285 min after injection was selected to compare cardiac MC1 density in HVs with that of participants with FA. Representative SUVR−1 images from a HV and a participant with FA are shown in Figure 4A. SUVR−1 values (Fig. 4B) were significantly lower in participants with FA than in HVs for all cardiac regions considered (*P* < 0.05). On average, SUVR−1 was 53% lower in the septum, 54% lower in the LVFW, 45% lower in the RVFW, and 54% lower in the combined septum and LVFW. Notably, no differences were observed in the volumes of the cardiac regions (data not shown; all *P* > 0.05). Free fraction in plasma (*f*_p_) was comparable between participants with FA (0.067 ± 0.008) and HVs (0.071 ± 0.013).

**FIGURE 5.**
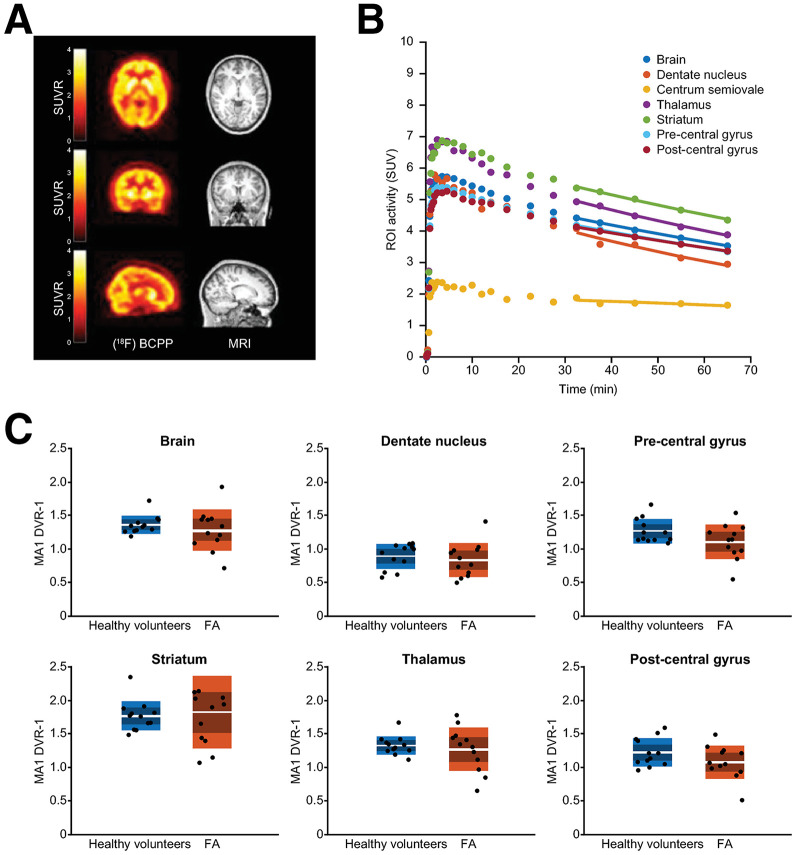
Comparison of brain MC1 density in HVs and participants with FA. (A) Orthogonal cross-section images of coregistered PET and MR images from individual with FA. PET images are shown as SUVR−1 (plasma) summed from 50 to 70 min. (B) SUV time–activity curves (circles) and multilinear analysis 1 fit (solid lines) in different brain regions for participant with FA. (C) Comparison of multilinear analysis 1 (MA1)–derived DVR−1 values between nonconcurrent HVs (blue) and participants with FA (red). DVR was calculated using centrum semiovale as pseudoreference region. ROI = region of interest.

Brain images were acquired from 12 participants with FA (Fig. 5A) and compared with data from nonconcurrent age- and sex-matched HVs from the MIND MAPS database (15). Kinetic modeling with the multilinear analysis 1 method produced acceptable model fits to tissue time–activity curves in all cases, and Figure 5B shows representative SUV time–activity curves and model fits from a participant with FA. *V*_T_ in participants with FA was 17%–25% lower than that in HVs in the dentate nucleus, thalamus, striatum, precentral gyrus, postcentral gyrus, and whole brain (*P* < 0.05 for all comparisons), and on average, there was a 21% reduction in *V*_T_ across the regions. Other than the dentate nucleus, which showed a 30% reduction in volume in participants with FA (*P* < 0.05), no brain regional volumetric differences were observed between HVs and participants with FA. The *f*_p_ was also 24% lower in participants with FA (0.067 ± 0.008) than in HVs from the MIND MAPS database (0.088 ± 0.022), whereas *V*_T_/*f*_p_ was not different. When considering distribution volume ratio−1 (DVR−1) values, a 13% reduction in participants with FA versus HVs was found in the precentral gyrus (*P* = 0.094; Fig. 5C). When considering SUVR−1, the pre- and postcentral gyrus showed 15% and 14% reductions, respectively, in participants with FA versus HVs, but neither was statistically significant (both *P* > 0.05) (Supplemental Fig. 3).

### Correlation Between Guanine–Adenine–Adenine (GAA) Expansion Size, Blood Frataxin, and MC1 Density in Participants with FA

**FIGURE 6.**
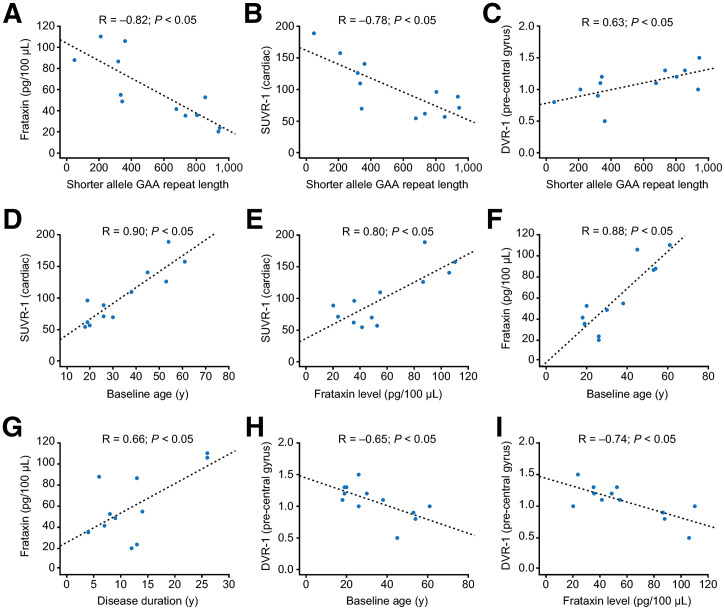
Relationships between GAA repeat length, cardiac, or brain MC1 density, and age, blood frataxin levels, or disease duration in participants with FA. Correlations between blood frataxin levels and shorter allele GAA repeat length (A); SUVR−1 in combined septum and LVFW and shorter allele GAA repeat length (B); DVR−1 in precentral gyrus and shorter allele repeat length (C); SUVR−1 in combined septum and LVFW and baseline age (D); SUVR−1 in combined septum and LVFW and baseline blood frataxin levels (E); baseline frataxin level and age at baseline (F); baseline frataxin level and disease duration (G); DVR−1 in precentral gyrus and baseline age (H); and DVR−1 in precentral gyrus and baseline blood frataxin level (I).

As expected from the established relationship between GAA repeat length of the shorter allele and frataxin expression, there was a negative linear correlation between the shorter allele repeat length and blood frataxin (*R* = −0.82; *P* < 0.05) (Fig. 6A). Similarly, SUVR−1 in the combined cardiac septum and LVFW region showed a negative correlation with repeat length in the shorter allele (*R* = −0.78; *P* < 0.05) (Fig. 6B). However, DVR−1 in the precentral gyrus showed a positive correlation with repeat length in the shorter allele (*R* = 0.63; *P* < 0.05) (Fig. 6C).

### Correlation Between MC1 Density and Baseline Clinical Characteristics of Participants with FA

Age at study baseline was strongly positively correlated with SUVR−1 in the combined cardiac septum and LVFW (*R* = 0.90; *P* < 0.05) (Fig. 6D). Similarly, SUVR−1 in that region was positively correlated with blood frataxin protein levels (*R* = 0.80; *P* < 0.05) (Fig. 6E). In addition, blood frataxin levels positively correlated with both age (*R* = 0.88; *P* < 0.05) (Fig. 6F) and disease duration (*R* = 0.66; *P* < 0.05) (Fig. 6G). In contrast, age at baseline was negatively correlated with DVR−1 in the precentral gyrus (*R* = −0.65; *P* < 0.05) (Fig. 6H), and DVR−1 in the precentral gyrus was also negatively correlated with blood frataxin protein levels (*R* = −0.74; *P* < 0.05) (Fig. 6I).

## DISCUSSION

This study of [^18^F]BCPP-EF–based PET imaging in a conditional cardiac and skeletal muscle knockout FA mouse model, HVs, and participants with FA demonstrates for the first time, to the best of our knowledge, that reduced MC1 density due to frataxin deficiency can be measured in vivo and shows correlation with GAA expansion size and some baseline clinical characteristics. Cardiac MC1 density in participants with FA was significantly lower than in HVs, and MC1 density in the precentral gyrus was 13% lower in participants with FA, although this was not statistically significant. Blood frataxin and cardiac [^18^F]BCPP-EF levels decreased with increasing GAA expansion size, but precentral gyrus [^18^F]BCPP-EF levels did not. Other correlation analyses showed that older participants with FA had higher cardiac MC1 density and higher blood frataxin levels compared with younger participants. By comparison, participants with FA who were older at baseline had reduced MC1 density in the precentral gyrus compared with younger participants, which was not correlated with blood frataxin levels. Similar reductions in MC1 density with age were observed in some brain regions (such as the caudate, thalamus, and hippocampus) in healthy individuals in a previous study (15).

The *f*_p_ of participants with FA was 24% lower than that in HVs from the MIND MAPS database. Since *f*_p_ drops out when calculating DVR, the main outcome parameter for the brain data, differences in *f*_p_ between participants with FA and HVs should not affect the brain data comparison. For cardiac data, the *f*_p_ values in participants with FA and HVs were similar, minimizing its impact on comparison.

We observed a positive correlation between DVR−1 in the precentral gyrus and the shorter GAA repeat length. However, this is at odds with preconceived assumptions on the relationship between GAA repeat length and MC1 in the brain. This may reflect that study participants were drawn from a sample of individuals with less severe FA who may not be representative of the broader FA population. Moreover, older patients are more likely to have shorter GAA repeat lengths, leading to milder disease, which provides one possible explanation (see also below). This finding warrants further study.

We also observed correlation between cardiac MC1 density and blood frataxin protein levels, suggesting PET measures of MC1 density can be used as a surrogate for frataxin levels, albeit not directly in the target organs. As noted above, however, the correlation analysis identified an apparent contradiction with increased frataxin levels observed with increasing age or increasing disease duration. The participants with FA were on average 34 y old at baseline (range, 18–61 y) and did not include pediatric individuals. Participants were selected to be ambulatory and had generally milder disease (scores on the Scale for the Assessment and Rating of Ataxia (24) ranged from 8 to 22, with a mean score of 15). Given the potential healthier baseline participant state and small study sample size, the study population may not be representative of the broader FA population. This finding may also be explained by the fact that the current study included a mixture of participants with early onset (i.e., classical) FA and late-onset FA. Patients with late-onset FA (e.g., age at onset ≥ 25 y) have a less severe phenotype than patients with an earlier onset (25), suggesting the MC1 estimates in younger patients are caused by more severe loss of frataxin compared with older patients. This is in line with prior studies that showed frataxin levels were positively correlated with age at onset and were higher in patients with late-onset FA compared with those with classic FA (26). One possibility is that older individuals in general have lower MC1 activity in the precentral gyrus and that the positive correlation with frataxin reported in this study (Fig. 6F) simply reflects the higher frataxin levels found in participants with late-onset FA. We observed a negative correlation not only between MC1 activity and age in the precentral gyrus of participants with FA (Fig. 6H) but also when MC1 activity versus age was examined in HVs (Supplemental Fig. 4). This may also be because patients with FA with very early onset, severe disease, and low frataxin levels (27) were not included in this study because of the inclusion and exclusion criteria. Further studies with patients stratified according to severity of disease would be needed to directly address this question.

This study had several limitations. The sample size in each group was small, and a nonconcurrent HV cohort (15) was used for comparison of brain imaging data. The study may have been underpowered to observe differences in brain MC1 density since a sample size of 20 participants was predicted to observe differences with 80% power. This observation should be taken forward for future studies. The original study protocol called for 12 HVs for cardiac imaging, but additional participants had to be recruited to optimize the procedure. Blood frataxin levels were used for the correlation analysis because of a lack of feasibility of directly measuring frataxin levels in the tissues of interest. As a result, the findings of these correlation analyses of some variables showing high variability limit generalization and warrant independent replication.

## CONCLUSION

We have identified [^18^F]BCPP-EF–based PET imaging of MC1 density as a possible biomarker in individuals with FA. This technique has the advantage of evaluating brain and cardiac MC1 density in a single visit using an optimized data acquisition protocol that does not require invasive tissue biopsy. Our findings suggest [^18^F]BCPP-EF–based PET imaging can be used repeatedly in patients with FA and could be used to monitor disease status and disease progression or as a possible endpoint in clinical trials on further validation and optimization.

## DISCLOSURE

Study funding was provided by Pfizer. Laigao Chen, Jeffrey Palmer, Avery McIntosh, Pengling Sun, and Lawrence Charnas are employees of and have stock or stock options in Pfizer Inc. Mark Aldridge, Mickael Huiban, Sara Moz, Jan Passchier, Lauren Sauvage, Rachel Stewart, Lisa Wells, and Eugenii Rabiner are employees of Invicro, a for-profit contract research organization. Christine Bulawa, Koene Van Dijk, Erica Henning, Alain Martelli, Marko Pregel, and Peter Loudon were employees of Pfizer for the duration of this study. Gaia Rizzo, Roger Gunn, and Allan Listanco were employees of Invicro for the duration of the study. No other potential conflict of interest relevant to this article was reported.

## Notes

KEY POINTS **QUESTION:** Can noninvasive PET imaging of MC1 density be used as a surrogate biomarker of frataxin in individuals with FA? **PERTINENT FINDINGS:** Using an optimized imaging protocol, MC1 density in the heart was lower in participants with FA than in HVs, a finding mirrored in a mouse model of FA. Correlation analyses showed that blood frataxin and cardiac MC1 density levels negatively correlated with GAA expansion size. **IMPLICATIONS FOR PATIENT CARE:** PET imaging of cardiac and brain MC1 density can be evaluated in a single visit using an optimized data acquisition protocol and is a possible biomarker in people with FA that requires further assessment and validation.
